# Karyotype changes in cultured human corneal endothelial cells

**Published:** 2008-05-19

**Authors:** Takashi Miyai, Yoko Maruyama, Yasuhiro Osakabe, Ryohei Nejima, Kazunori Miyata, Shiro Amano

**Affiliations:** 1 Miyata Eye Hospital, Miyakonojo, Japan; 2 Department of Ophthalmology, University of Tokyo Graduate School of Medicine, Tokyo, Japan

## Abstract

**Purpose:**

To examine karyotype changes in cultured human corneal endothelial cells (HCECs).

**Methods:**

HCECs with Descemet’s membrane were removed from 20 donors of various ages (range, 2–77 years; average, 43.7±26.4 years) and cultured on dishes coated with extracellular matrix produced by bovine corneal endothelial cells (BCECs). Karyotype changes were examined by G-band karyotyping of HCECs at the third passage from 12 donors and the fifth passage from 16 donors. The number of chromosomes was analyzed in 40–50 cells from the third and fifth passages of each HCEC preparation. A detailed karyotype analysis of 10–16 cells from the third and fifth passages of each HCEC preparation was performed. The frequency of aneuploid cells per case (the number of abnormal cells divided by the total number of cells examined at metaphase) was tested for correlation with age by Spearman’s correlation analysis.

**Results:**

At the third passage, five cases (41.7%) showed an almost normal karyotype, and five cases (41.7%) showed sex chromosome loss. One case (8.3%) showed chromosome 21 trisomy. At the fifth passage, five cases (31.3%) showed an almost normal karyotype, and four cases (25%) showed sex chromosome loss. Three cases (18.8%) showed chromosome 8 trisomy, and one case (6.3%) showed chromosome 21 trisomy. Donor age and the frequency of aneuploidy had a statistically significant correlation at the fifth passage (R=0.653, p=0.042).

**Conclusions:**

Donor age and the frequency of aneuploidy have a positive correlation in cultured HCECs at the fifth passage. Therefore, HCECs for clinical therapies should be obtained from donors as young as possible. Karyotyping cultured HCECs is crucial before clinical application.

## Introduction

Corneal endothelial dysfunction is caused by various diseases and surgeries such as Fuchs’ endothelial dystrophy, trauma, cataract surgery, glaucoma surgery, and laser iridotomy and is a major cause of corneal transplantation. Because corneas from donors are in grave shortage worldwide, there is a potentially large role for corneal endothelial tissue bioengineering in treating patients with endothelial dysfunction. Since human corneal endothelial cells (HCECs) can be grown in culture [1-7], the application of cultured HCEC transplantation to corneal endothelial dysfunction has been investigated [8-13]. However, cultured cells tend to have a potential risk of karyotype changes [14,15], which are often associated with carcinogenesis. Thus, cultured HCECs should be carefully examined before clinical application. Moreover, preparing and examining cultured HCECs on time for surgery could be difficult since the time of the donor’s death cannot be predicted accurately before the donor’s death. Cryopreservation of cultured HCECs provides a solution for this problem if its safety can be proven. We conducted this study to examine karyotype changes in cultured HCECs with or without cryopreservation.

## Methods

### Human corneal endothelial cell donors

HCECs were obtained from 20 human cadaver corneas and were cultured before performing karyotyping analysis. The donor age ranged from 2 to 75 years (average 43.7±26.4). Nine males and 11 females were included. All donor corneas were preserved in Optisol GS (Bausch & Lomb, Rochester, NY) and imported by airplane from the Rocky Mountain Lions Eye Bank for research purposes. The donor information showed that all donor corneas were considered healthy without corneal disease and all donors had no past history of chromosomal abnormality. The demographic data of the donors are shown in Table 1. The confidentiality of donor information was maintained according to the Declaration of Helsinki.

**Table 1:** Demographic data of donors.

**Donor#**	**Age**	**Gender**	**Cause of death**	**Ventilation**	**Death to preservation time**	**Death to culture time**
1	3	F	Medulloblastoma	Yes (1 month)	5:59	9 days
2	12	M	Anaplastic astrocytoma	No	12:06	10 days
3	21	F	Seizure disorder	No	15:58	6 days
4	38	F	Lymphangioleiomyomatosis	Yes (1 month)	4:41	5 days
5	55	M	Myocardial infarction	No	7:23	11 days
6	60	F	Colon cancer	NA	3:10	2 days
7	61	M	Pancreatic cancer	No	7:15	8 days
8	73	F	Chronic obstructive pulmonary disease	No	4:49	6 days
9	18	M	Trauma	Yes (20 min)	13:12	7 days
10	57	M	Myocardial infarction	Yes (24 h)	4:35	8 days
11	70	M	Myocardial infarction	Yes (7 h)	5:34	6 days
12	77	M	Cerebrovascular accident	Yes (7 h)	3:36	8 days
13	2	M	NA	NA	NA	NA
14	4	F	Congestive heart failure	No	9:10	7 days
15	20	F	NA	NA	NA	NA
16	45	F	Colon cancer	NA	2:00	5 days
17	57	F	GI bleed	No	3:43	7 days
18	58	M	Renal disease	No	5:00	5 days
19	68	F	Bowel cancer	Yes (20 days)	6:16	6 days
20	75	F	Renal failure	No	5:29	8 days

The table has been arranged according to donor age in three subgroups as follows: #1–8, the examination was conducted at both passages 3 and 5; #9–12, the examination was conducted at passage 3 only; and #13–20, the examination was conducted at passage 5 only. Death to preservation time is expressed in hours and minutes. “NA” denotes that data were not available.

### Preparation of culture dishes

All culture dishes were coated with bovine corneal endothelial extracellular matrix according to a previously reported method [7]. Bovine corneal endothelial cells were cultured from young, locally obtained calf eyes. Primary cultures of bovine corneal endothelial cells (BCECs) were established from explants of the corneal endothelial cell layer in 60 mm culture dishes after which they were propagated using standard techniques in a medium consisting of Dulbecco’s modified Eagle’s medium with 10% fetal bovine serum, 5% calf serum, 30 mg/l of L-glutamine, 2.5 mg/l of fungizone, 2.5 mg/l of doxycycline, 2 ng/ml of basic fibroblast growth factor, and 2% dextran. For extracellular matrix (ECM) production, six confluent 100 mm dishes of BCECs were trypsinized using trypsin-EDTA (0.05% trypsin and 0.53mM of EDTA) and then added to 500 ml of the ECM production media. Unless otherwise specified, the media were changed every two to three days for HCEC and BCEC cultures. The cell suspension was added at volumes appropriate for the culture dishes being prepared and then was incubated until confluent without a media change. Cells were then lysed for 5 min in a 0.14% ammonium hydroxide solution in distilled water. The plates were rinsed three times in Ca^2+^-free and Mg^2+^-free phosphate buffered saline. After having been scanned by microscope to confirm complete removal of all the bovine corneal endothelial cells, the dishes were stored at 4 °C in a sterile medium consisting of Ca^2+^-free and Mg^2+^-free phosphate buffered saline with 2.5 mg/l of fungizone and 12.5 mg/l of doxycycline until use in culture.

### Human corneal endothelial cell culture

HCEC culture was prepared as described in our previous report [7]. Primary cultures were established from explants of the endothelial cell layer with Descemet’s membrane from the peripheral part of the cornea and were propagated on the culture dishes coated with bovine corneal endothelial extracellular matrix. HCECs were cultivated with Dulbecco’s modified Eagle’s medium supplemented with 15% fetal bovine serum, 30 mg/l of L-glutamine, 2.5 mg/l of fungizone (GIBCO BRL, Grand Island, NY), 2.5 mg of doxycycline, and 2 ng/ml of basic fibroblast growth factor. Cell lines were maintained in a humidified incubator at 37 °C in 10% CO_2_. The media were changed every two or three days. After confirming adequate density of proliferating cells after about 10–20 days, passaging procedure was performed. HCECs were rinsed three times in Ca^2+^-free and Mg^2+^-free phosphate buffered saline, trypsinized for 2 min at 37 °C, and passaged at ratios of 1:4. All subsequent passaging was performed using the same method after HCECs in the dishes became confluent. The approximate time to confluence after each passaging was six to eight days.

### Cryopreservation

Several cell lines were processed for cryopreservation. Third passage HCECs were frozen by mixing them with a cell freezing solution (Cell Banker®; Juji Field Inc, Tokyo, Japan) and preserved at −152 °C. The frozen HCECs were defrosted in a 37 °C homeothermal tank. After adding 10 ml of culturing solution, defrosted HCECs were centrifuged and plated into dishes.

### Karyotyping

Cytogenetic examination was performed at the third passage from 12 donors and at the fifth passage from 16 donors. In eight donors, cytogenetic examination was performed at both the third and fifth passages. In the early stage of this study, cytogenetic examination was performed only at the fifth passage. Moreover, HCECs from several donors could be cultured to the third passage but not to the fifth passage. These are the reasons why there are unequal sample sizes for the third and fifth passages. Standard cytogenetic harvesting, fixation, and G-bands after trypsin and Giemsa (GTG banding) techniques were used for HCECs. After incubation with 0.06 μg/ml colcemid for 16 h to arrest cell mitosis, HCECs were detached using 0.05% trypsin/EDTA. HCECs were treated with 0.075 M KCl and then fixated with Carnoy’s fixative (a 3:1 mixture of methanol and glacial acetic). HCEC solution was dropped onto a slide glass and air-dried. Then, HCECs were treated with 0.005% trypsin for 7 min and stained with 6% Giemsa stain solution for 3.5 min. The number of chromosomes was analyzed in 40–50 cells from the third and the fifth passages of each HCEC preparation. A detailed karyotype was analyzed in 10–16 cells from the third and the fifth passages of each HCEC preparation. The standard International System for Human Cytogenetic Nomenclature (ISCN), 1995, and definitions were followed.

### Statistics

The frequency of loss or gain of individual chromosomes was examined. The frequency of aneuploidy per case (the number of abnormal cells divided by the total number of cells examined at metaphase) was tested for correlation with age by Spearman’s correlation analysis. Stepwise multiple regression analysis was performed to investigate the relationship between two variables (donor age and presence of cryopreservation) and the frequency of aneuploidy. We considered p values <0.05 as statistically significant.

## Results

**Figure 1:**
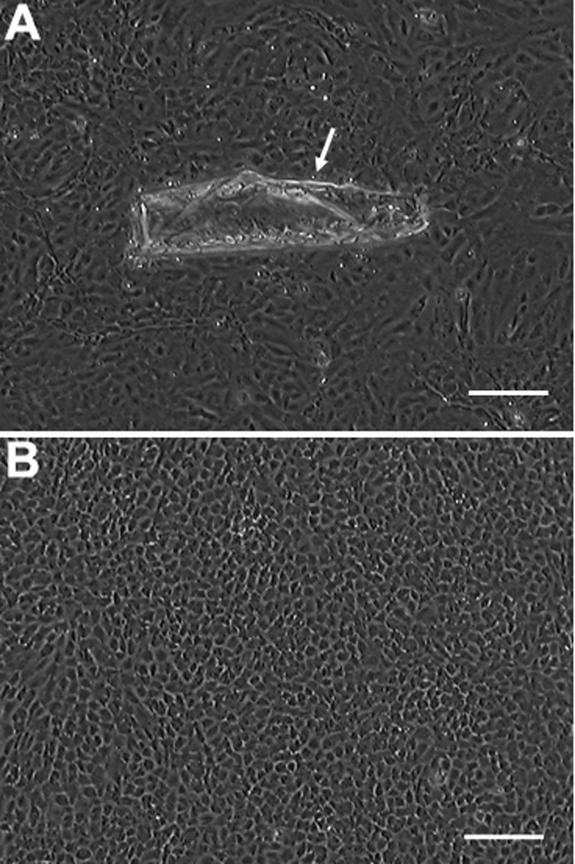
Examples of HCEC cultures. Cultured HCECs at primary culture (**A**) and fifth passage (**B**). **A**: The donor age was five years old. **B**: The donor age was 21 years old. Hexagonal confluent cells were recognized in both cultures. A strip of Descemet’s membrane (arrow in **A**) was observed in the primary culture. Bars=100 μm

**Figure 2:**
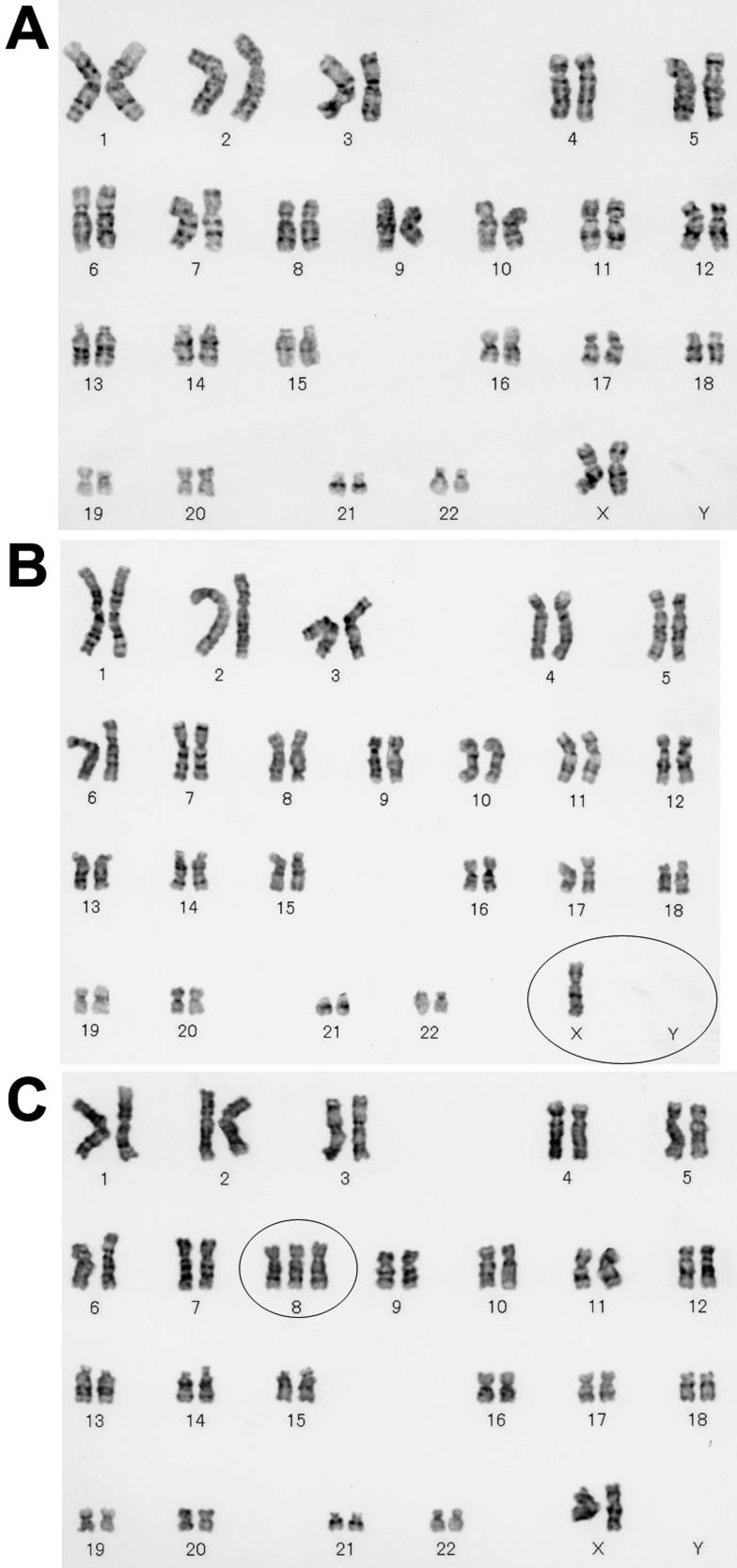
Examples of karyotypes of cultured human corneal endothelial cells. **A**:Normal karyotype (46, XX) was observed in passage 5 of 60-year-old donor (#6). **B**: Sex chromosome Y deficiency (45, X, –Y) was observed in passage 5 of 61-year-old donor (#7). A circle shows the deficiency of sex chromosome Y. **C**: Chromosome 8 trisomy (47, XX, +8) was observed in passage 3 of 38-year-old donor (#4). A circle shows chromosome 8 trisomy.

Figure 1 shows the examples of HCECs of young and old donors at primary culture and fifth passage. HCECs could be cultured to be confluent, and hexagonal confluent cells were recognized at both primary and fifth passages. Figure 2 shows representative examples of cultured HCEC karyotypes.

Table 2 shows the distribution of the total chromosome number and aneuploidy frequency of cultured HCECs at the third passage, and Table 3 shows the distribution at the fifth passage. The frequency of aneuploidy is expressed as a percentage. All examined cells showed the normal number of chromosomes in 4 out of 12 cases at the third passage and in 2 out of 16 cases at the fifth passage.

**Table 2:** Distribution of total chromosome number and the frequency of aneuploidy in cultured human corneal endothelial cells at the third passage.

**Donor #**	**Age**	**Cryopreservation**	**Number of chromosomes**						**Frequency of aneuploidy (%)**
			**42**	**43**	**44**	**45**	**46**	**47**	
1	3	-	0	0	0	0	50	0	0
2	12	-	0	0	0	0	50	0	0
3	21	-	0	0	0	0	49	1	2
4	38	+ (28 months)	0	0	0	0	43	6	12.2
5	55	+ (26 months)	0	0	0	42	6	2	88
6	60	-	0	0	0	3	45	2	10
7	61	+ (25 months)	0	0	0	43	7	0	86
8	73	-	0	0	0	7	18	25	64
9	18	+ (23 months)	0	0	0	0	50	0	0
10	57	-	0	0	0	50	0	0	100
11	70	-	0	0	3	1	43	3	14
12	77	+ (13 months)	0	0	0	0	50	0	0

Each subcolumn within the “Number of chromosomes” column shows the number of cells found to be containing the specific number of chromosomes corresponding to the subcolumn heading. This table has been arranged according to donor age in 2 subgroups as follows: #1~8: The examination was conducted at both passages 3 and 5. #9~12: The examination was conducted at passage 3 only.

**Table 3:** Distribution of total chromosome number and the frequency of aneuploidy in cultured human corneal endothelial cells at the fifth passage.

**Donor #**	**Age**	**Cryopreservation**	**Number of chromosomes**						**Frequency of aneuploidy (%)**
			**42**	**43**	**44**	**45**	**46**	**47**	
1	3	-	0	0	0	2	48	0	4
2	12	-	0	0	0	0	50	0	0
3	21	-	0	0	1	0	49	0	2
4	38	+ (28 months)	0	0	0	0	41	9	18
5	55	+ (26 months)	0	0	0	48	0	2	100
6	60	-	0	0	0	0	50	0	0
7	61	+ (25 months)	0	0	0	50	0	0	100
8	73	-	0	0	1	0	5	44	90
13	2	+ (period n.a.)	0	0	1	6	25	9	39
14	4	+ (period n.a.)	0	1	1	2	40	1	11.1
15	20	+ (period n.a.)	0	0	0	1	45	4	10
16	45	+ (4 months)	1	1	1	0	6	41	88
17	57	+ (16 months)	0	0	1	2	46	1	8
18	58	+ (1 month)	0	1	2	29	18	0	64
19	68	+ (17 months)	0	1	1	48	0	0	100
20	75	+ (19 months)	0	0	0	1	14	35	72

Each subcolumn within the “Number of chromosomes” column shows the number of cells found to be containing the specific number of chromosomes corresponding to the subcolumn heading. This table has been arranged according to donor age in 2 subgroups as follows: #1~8: The examination was conducted at both passages 3 and 5. #13~20: The examination was conducted at passage 5 only.

Table 4 and Table 5 present detailed karyotyping of the third and fifth passages, respectively. At the third passage, five cases (#1, 2, 3, 9, 12) showed almost normal karyotypes whereas five cases (#5, 6, 7, 10, 11) showed sex chromosome loss. One case (#8) showed chromosome 21 trisomy. At the fifth passage, five cases (#1, 2, 3, 6, 14) showed almost normal karyotypes while four cases (#5, 7, 18, 19) showed sex chromosome loss. Three cases (#15, 16, 20) showed chromosome 8 trisomy, and one case (#8) showed chromosome 21 trisomy. In eight cases (#1–8), karyotyping was examined at both the third and fifth passages. When the changes in karyotyping from the third to fifth passages were analyzed in these cases, a normal karyotype was maintained in three cases (#2, 3, 6) and the same abnormal karyotype was maintained in four cases (#4, 5, 7, 8). In case #1, the frequency of abnormal karyotype (45, X, -X) increased from the third passage to the fifth passage.

**Table 4:** Karyotyping of human corneal endothelial cells at the third passage.

**Donor #**	**Karyotyping**
1	46,XX,t(12;18)(q13;121)=1,46XX=10
2	46,XY=10
3	46XX,?inv(2)(p11q12),t(6;11)(q23;p13)=1,47,XX,+3=1, 46,XX=10
4	47,XX+3=2, 48,idem,+21=1, 47,XX,+X=1, 47,XX,+8=1, 47,XX,+12=1, 47,XX,+21=1, 46,XX=9
5	45,X,-Y=10, 47,idem,+7,+8=2, 46,idem,+X=1, 46,idem,+8=1, 46,XY=2
6	45,X,-X=3,47,XX,+20=1,47,XX,+21=1,46,XX=10
7	45,X,–Y=7, 46,idem,+8=3
8	47,XX,+21=5, 45,X,-X=2,46,XX=3
9	46,XY=10
10	45,X,-Y=10
11	45,X,-Y=7, 43,X,-Y,-21,-22=1 45,X,-Y,-11,+20=1 46,X,-Y,-14,+16,+20=1
12	46,XY=10

Specific karyotypes found in each donor and cell number in which each specific karyotype was found are shown.

**Table 5:** Karyotyping of human corneal endothelial cells at the fifth passage.

**Donor #**	**Karyotyping**
1	45,X,-X=2,46,XX,t(2;17)(p10;q10)=1,46XX=10
2	46,XY=10
3	44,XX,-19,-22=1, 46,XX=10
4	47,XX,+X=4, 47,XX,+3=2, 46, XX=10
5	45,X,-Y=10, 47,idem,+7,+8=1, 47,XY,+20=1
6	46XX=10
7	45,X,-Y=10
8	47,XX,+21=10 46,XX=1
13	45,X,-Y=2, 45,idem,t(1;14)(p34;p13)=1,47,XY,+18=3,46,XY=6
14	46XX=10
15	47,XX,+8=4,46,XX=10
16	47,XX,+8=10,46,XX,=3
17	46,X,-X,+8=5,47,XX,+4=1,46,XX=4
18	45,X,-Y=4 46,idem,+3=1 46XY=5
19	45,X,-X=10
20	47,XX,+8=10

Specific karyotypes found in each donor and cell number in which each specific karyotype was found are shown.

**Figure 3:**
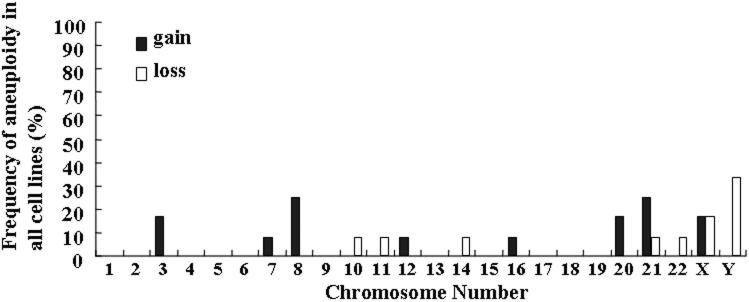
Distribution of chromosome aneuploidy in cultured human corneal endothelial cells at third passage. The frequency of gain or loss of each chromosome is shown.

**Figure 4:**
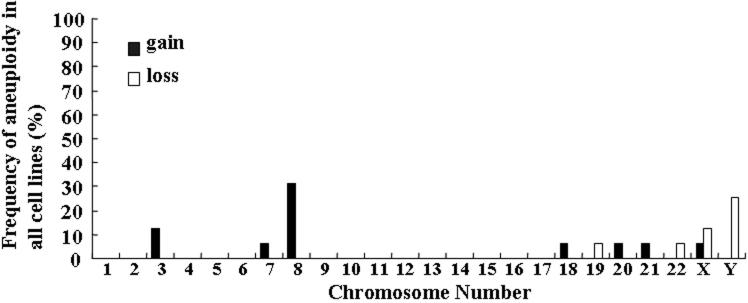
Distribution of chromosome aneuploidy in cultured human corneal endothelial cells at fifth passage. The frequency of gain or loss of each chromosome is shown.

Figure 3 and Figure 4 present the histograms of the distribution of chromosome aneuploidy that was observed in cultured HCECs at the third and fifth passages. At the third passage, 25% of the cases gained chromosome 8 and 33.3% of the cases lost chromosome Y. At the fifth passage, 31.3% of the cases gained chromosome 8 and 25% of the cases lost chromosome Y.

**Figure 5:**
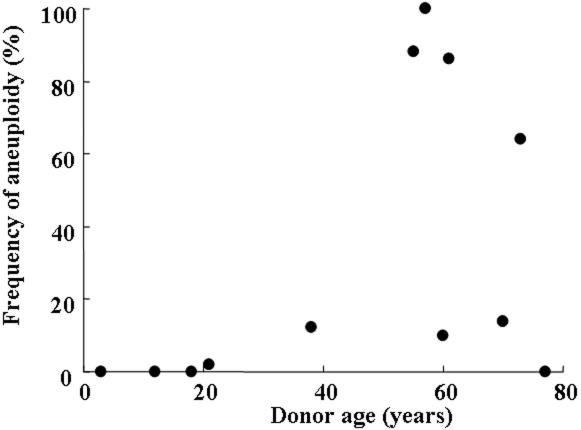
Relationship between donor age and the frequency of aneuploidy in cultured human corneal endothelial cells at the third passage. Donor age and the frequency of aneuploidy had no significant correlation (R=0.437, p=0.147).

**Figure 6:**
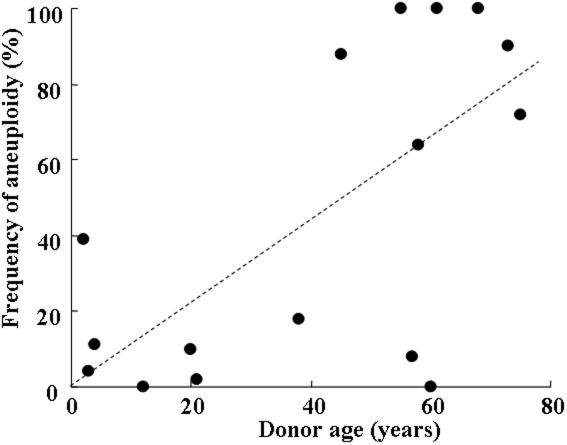
Relationship between donor age and the frequency of aneuploidy in cultured human corneal endothelial cells at the fifth passage. Donor age and the frequency of aneuploidy had a statistically significant correlation (R=0.653, p=0.042). The equation of the regression curve is: (frequency of aneuploidy) = 2.08+1.032 x (age).

Figure 5 and Figure 6 show the relation between donor age and the frequency of aneuploidy at the third and fifth passages, respectively. Donor age and the frequency of aneuploidy had no significant correlation at the third passage (R=0.437, p=0.147) but had a statistically significant correlation at the fifth passage (R=0.653, p=0.042). The regression curve in the fifth passage is expressed by the equation:

(frequency of aneuploidy) = 2.08+1.032 x (age).

Stepwise multiple regression analysis revealed that age was the only explanatory variable that was relevant to the frequency of aneuploidy (R=0.660, p=0.0029). The other variable, cryopreservation, was irrelevant to the frequency of aneuploidy.

## Discussion

In the current study, cultured HCECs demonstrated aneuploidy at the third and fifth passages in most cases. Has the aneuploidy observed in cultured HCECs been present in vivo or has it been induced during culture? Aneuploidy is induced during cell division, and HCECs hardly show cell division in vivo. Thus, the aneuploidy observed in cultured HCECs may have been induced during culture. However, because cells need to go into the cell division cycle when we examine the karyotype using our method, we could not investigate the karyotype before culture. Changes in karyotype from the third to fifth passage were examined in eight cases. The same karyotype was maintained in seven of those cases, and a new abnormal karyotype appeared in only one of them. Taken together, most abnormal karyotypes observed in cultured HCECs may have been induced at a very early stage during culture.

Donor age and the frequency of aneuploidy had a significantly positive correlation at the fifth passage. This result suggests that chromosome damage accumulates in HCECs in vivo with increasing age and that aneuploidy is induced in HCECs when they are forced to undergo cell division in vitro. Previous studies reported that the telomere is not shortened in HCECs of elderly persons probably due to the very low proliferative activity of HCECs in vivo [16,17]. Thus, the shortening of telomeres is not a cumulative change in HCECs with increasing age. Further studies are necessary to elucidate the specific mechanisms of accumulating damage in HCEC chromosomes with increasing age.

In our study, cultured HCECs tended to have a chromosome mosaic of sex chromosome monosomy and chromosome 8 trisomy. Previous studies reported that the X chromosome in females and the Y chromosome in males showed age-dependent loss in peripheral lymphocytes [18-21], bone marrow cells [21], and corneal keratocytes [22,23]. Thus, loss of sex chromosomes is most frequently observed as an age-dependent chromosome abnormality regardless of cellular type. The results in this study confirmed that the loss of a sex chromosome tends to occur in HCECs with increasing age as in other types of cells. On the other hand, chromosome 8 trisomy was the most frequently observed trisomy in acute myeloid leukemia [24], and chromosome 8 trisomy mosaic syndrome is associated with corneal opacity [25]. In addition, chromosome 8 includes oncogenes *c-myc* and *c-mos*. Further study is necessary to elucidate the association between this information on chromosome 8 and the frequent occurrence of chromosome 8 trisomy in cultured HCECs.

Stepwise multiple regression analysis revealed that age but not cryopreservation was the explanatory variable relevant to the frequency of aneuploidy, suggesting that cryopreservation is irrelevant to aneuploidy. However, because the number of subjects without cryopreservation in this study was small, a further study with a larger number of cases with or without cryopreservation is needed to confirm the relationship between cryopreservation and aneuploidy.

Our previous study demonstrated that the average area of HCECs at the fourth passage as well as the percentage of cells larger than 2000 μm^2^ increased in a manner proportional to donor age [7]. Senoo et al. [26] reported that HCECs from old donors can proliferate but respond more slowly and to a lesser extent than cells from young donors. Moreover, HCECs from the central corneal area have a relatively lower replicative competence in older donors than in young donors [27]. These age-related reductions in relative proliferative capacity and the senescence characteristics previously observed in cultured HCECs may be related to the chromosomal abnormalities observed in this study.

Because karyotype changes are often associated with carcinogenesis, the existence of chromosomal abnormalities in HCECs is clearly a concern for clinical use. The results in this study demonstrated that donor age and frequency of aneuploidy have a significantly positive correlation at the fifth passage. Therefore, our results indicate that HCECs for clinical therapies should be obtained from donors as young as possible. Moreover, careful examination of karyotype in cultured HCECs is crucial before clinical application.
